# Prognostic value of non-invasive serum Cytokeratin 18 detection in gastrointestinal cancer: a meta-analysis

**DOI:** 10.7150/jca.31408

**Published:** 2019-08-27

**Authors:** Yuejuan Huang, Ling Yang, Yan Lin, Xin Chang, Huini Wu, Ying Chen

**Affiliations:** 1 Department of Chemotherapy, the People's Hospital of Baise City, No 8 Chengxiang Road, Baise, Guangxi 533000, People's Republic of China; 2 Affiliated Tumor Hospital of Guangxi Medical University, No 71 Hedi Road, Nanning, Guangxi 530021, People's Republic of China; 3 Department of Cell and Molecular Physiology, Loyola University Chicago, 2160 S. 1St Ave, Maywood, IL 60153, USA

**Keywords:** Cancer, Prognosis, Cytokeratins

## Abstract

**Background**: Gastrointestinal cancer is one of the most common neoplasms. Cytokeratin 18(CK18) is widely expressed in many different organs and cancers. Emerging data suggested conflicting results about the role of CK18 during carcinogenesis. The aim of this study is to systematically review the prognostic value of circulating CK18 (M65) and caspase-Cleaved CK18 (M30) in digestive cancers.

**Materials and Methods**: We searched major database for manuscripts reporting the effect of pretreatment CK18 on survival of digestive cancer patients. Revman5.3 and R were the software used for analysis. Pooled multivariable-adjusted hazard ratios (HR) for overall survival (OS) were calculated in all patients and many different subgroup analyses by stratifying on tumor type, metastasis stage, and ethnicity.

**Results**: 11 original studies were included for analysis. A low level of M30 and M65 were shown to be a protective factor for all cancer patients (HR 0.49, 95%CI 0.33-0.73, P=0.0003; HR 0.48, 95% CI 0.32-0.70, P =0.0001, respectively). The low M30 remained to be a protective factor for metastasized cancer patients while M65 had no statistically significant correlation with prognosis.

**Conclusions**: Non-invasive total and cleaved CK18 level detection by ELISA could be potentially a useful predictor of prognosis of digestive cancer patients. Further studies are warranted to investigate the molecular mechanisms of CK18.

## Introduction

Gastrointestinal cancer is one of the most common neoplasms in the world, accounting for nearly one third of total cancer cases. In developing countries, especially China, the incidence is even higher 1. Because of its lack of clinical signs at early stages, many patients were diagnosed at advanced stages with poor prognosis. Nowadays, tumor, lymph node, and metastasis (TNM) staging system and histological subtype are the most globally recognized clinicopathological variables for standard treatment and prognostication of gastrointestinal cancer 2. However, large variations in clinical outcomes have been observed in patients with the same staging and treatments 3-5. It suggests that current tumor staging system failed to include enough prognostic information for prediction and requires further improvements. Several novel biomarkers have shown good supplements to the current staging system 6-8. Many potential molecular predictors of prognosis, like cytokeratins, are controversial and need more investigation.

Cytokeratins are a conserved group of protein, which form cytoplasmic structure of epithelial cells and tissues. The expression pattern of cytokeratins is determined by different type of epithelial cells and differentiation. Cytokeratin 18 gene is located on chromosome 12q13, encoding a member of type 1 cytokeratins. Cytokeratin 18 is primarily expressed in single layer epithelial tissues. It binds with cytokeratin 9, a type two cytokeratin, to form heteropolymers in keratin filaments 9. It is widely expressed in a large number of different organs and cancers that arise from these tissues, including liver, pancreas, gastrointestinal tract, lung, breast and kidney 10. Cytokeratin 18 is involved in many cellular processes, such as apoptosis, mitosis, proliferation, cell cycle progression and responses to stresses 11-15. Importantly, CK18 is cleaved by Caspase 2 protein at Asp 396 during apoptosis. It could be detected by M30 ELISA assay in plasma to reflect tumor cell apoptosis intensity 16. On the other hand, M65 ELISA assay is used to determine the levels of both full-length CK18 protein and its fragments in plasma, monitoring both apoptosis and necrosis of tumor cells 16. Previously, conflicting results has been reported about the role of CK18 during carcinogenesis. Thus, it is necessary to conduct a meta-analysis to systematically study the prognostic values of serum total and cleaved CK18 in digestive cancers.

## Material and Methods

This meta-analysis was conducted according to the recommendations of the Preferred Reporting Items for Systematic Reviews and Meta-analysis (PRISMA) guideline (Supplementary Table 1).

### Literature search

We searched English-written eligible manuscripts independently in PubMed Central, Scopus and Web of Science. The last search was performed on May 1st, 2019. We used the following keywords and their combination in the searching: “cancer” or “tumor” or Carcinoma" or "neoplasm" or “malignancy”, and “prognosis”, and “cytokeratin (CK) 18”, or “M30”, or “M65” from 2008 January to 2018 August. All eligible manuscripts and their references were retrieved for further analysis.

### Selection criteria

All English-written manuscripts are collected according to the following criteria: (1) all cancers, including gastric cancer, hepatocellular carcinoma (HCC), colorectal cancer, and pancreatic cancer, all patients were histopathological diagnosed; (2) each study detected the levels of CK18 prior to the surgery; (3) hazard ratios (HRs) with their 95% confidence interval (95% CI) of CK18 in patients were available in the studies; (4) only the most recent study was included if the same investigator publish multiple studies using the same dataset.

### Quality assessment

We assessed the quality of each of the studies in this meta-analysis, adapted from Yan Lin, *et al*. 17. We included the following quality items in the assessment: whether or not a clear description of the objectives, whether or not including a clear ethical statement, whether or not a clear statement of the study period, whether or not a clear description of tumor stage, whether or not stating the patient selection criteria, whether or not stating the method of the CK18 cutoff in the study, whether or not define Disease free survival (DFS) /Cancer specific survival (CSS) /Overall survival (OS) prior to the results, whether or not use multivariate analysis and/or univariate analysis, whether or not a clear HR with 95% CI stated, whether or not limitations considered in the study. We ranked the included papers according to the quality items used in each study (score range 0-10). Quality assessment was not used as exclusion criterion for the eligible studies.

### Data extraction

The following information were extracted from each study: author, year of publication, country, study period, cancer type, stage of cancer, clinical setting, number of patients, male patient percentage, average age, follow up months, M30/M65 detection methods, cutoff, HR with its 95% CI.

### Statistical Analysis

We used Revman5.3 (The Nordic Cochrane Centre, the Cochrane Collaboration, Copenhagen, Denmark) and R programing language to further analyze data. During the full-paper screening process, inter-reviewer agreement was estimated by Cohen's kappa statistic. The cohort-specific HRs and their 95% CI from Cox hazard models were extracted from each study. Subgroup analyses were also used to study how cancer stages, races, age, and sex would affect M30/ M65 predictive effect of cancer patients.

We used Begg's funnel plot in Revman5.3 and Egger's test in R to analyze the publication bias. The packages meta, metagen and metaphor in R were used in this study. Sensitivity test was also performed by omitting each study to find potential outliers in R. Statistical heterogeneity between studies was determined by Cochran's Q test and Higgins I square. *P* < 0.1 or I^2^ > 50% was considered as heterogeneity. When there was no statistically significant heterogeneity, we used a fixed-effect model to combine the data. Otherwise, we used a random-effects model. A two-sided P value less than 0.05 was considered statistically significant.

## Results

### Literature search

**Figure 1:**
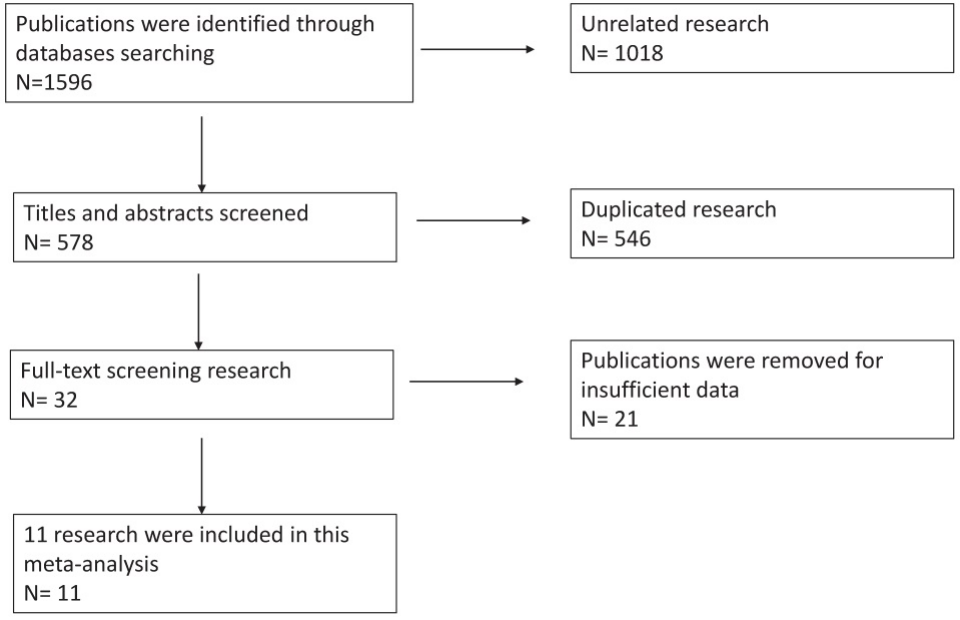
Flow-diagram of this meta-analysis.

We had a total of 1596 publications in the initial literature search. 32 were included in the full-text screening after scanning the titles and abstracts. We reviewed the including papers carefully and 21 publications were excluded for duplications or insufficient data. Eventually, 11 publications were included in this meta-analysis. Cohen's kappa for inter-reviewer agreement was 0.78 for first-stage screening and 0.82 for second-stage full-text screening. Our literature search flow chart was shown as Figure 1. The quality of the 11 included publications was good with an average quality score of 6.8 and a median score of 7 (range 4-9, Supplementary Table 2).

### Literature details

Overall, 1233 patients were included in this study. 5 (45.5 %) out of 11 studies were published after 2013 and 6 (54.5%) were before 2013. 7 (63.6%) of the studies assessed patients from west Europe, 1 (9.1 %) from east Asia, 3 (27.3 %) from Turkey. 8 (72.7 %) of the studies provided data of M30 expression, and 8 (72.7%) of the studies provided data of M65 expression, while 5 (45.5 %) of them provided data of both. Four (36.4 %) of the studies were in gastric cancer 18-21, two (18.2 %) studies in HCC 22, 23, three (27.3 %) studies in colorectal cancer 24-26, and two (18.2 %) in pancreatic cancer 27, 28. The cut-off value of M30 and M65 varies in different studies, ranging from 59.1 U/L to 887 U/L, and from 199.3 U/L to1614 U/L, respectively. The main characteristics of the 11 included studies were summarized in Table 1.

### Meta-analysis results

**Figure 2:**
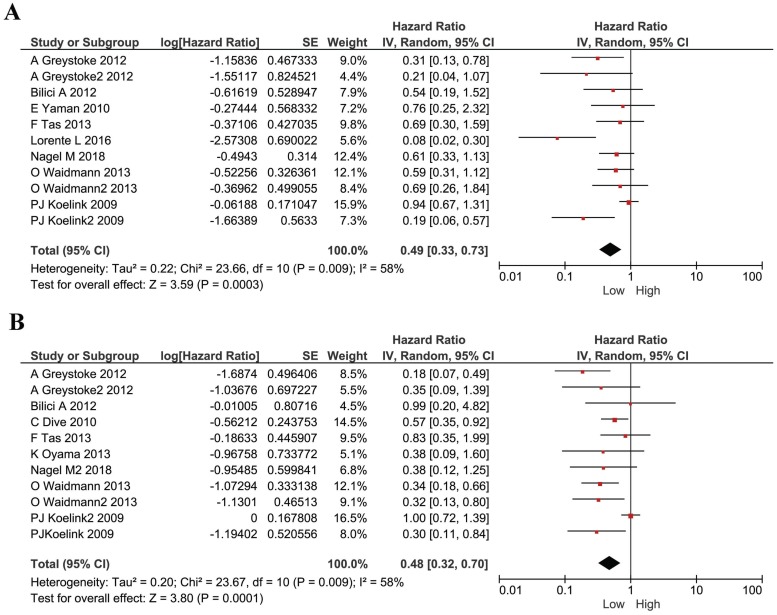
Forest plots displaying pooled hazard ratios (HRs) for survival in 11 studies. (A) the pooled hazard ratios (HRs) for M30 in all cancers; (B) the pooled hazard ratios (HRs) for M65 in all cancers.

We performed analyses to evaluate the prognostic effect of M30 and M65 in cancers. We used the HRs and their 95% CI in each study to calculate a combined HR. The estimated proportion of heterogeneity (I^2^) between M30 studies was 58% (*P*=0.0009) and so a random model was used. A low level of M30 was shown to be a protective factor for all cancer patients (HR 0.49, 95%CI 0.33-0.73, *P*=0.0003, Figure 2A). The estimated proportion of heterogeneity (I^2^) between all M65 studies was 58% (*P*= 0.0009) and we used a random model to combine all HRs. Similarly, cancer patients with a lower plasma M65 had a better survival (HR 0.48, 95% CI 0.32-0.70, *P* =0.0001, Figure 2B).

**Figure 3:**
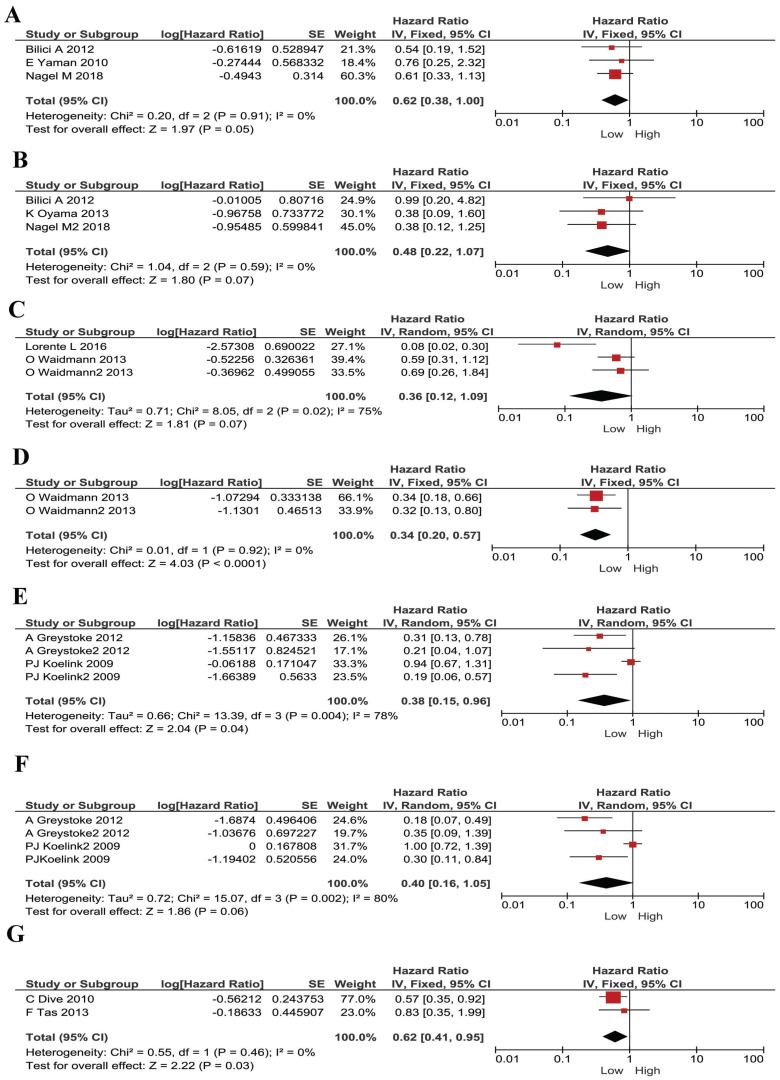
Forest plots displaying pooled hazard ratios (HRs) for patients stratified by cancer types in M30 and M65 groups. (A) The pooled HRs for M30 in patients with gastric cancers. (B) The pooled HRs for M65 in patients with gastric cancers. (C) The pooled HRs for M30 in patients with HCC. (D) The pooled HRs for M65 in patients with HCC. (E) The pooled HRs for M30 in patients with CRC. (F) The pooled HRs for M65 in patients with CRC. (G) The pooled HRs for M65 in patients with pancreatic cancer.

We understand each cancer has its own specificity. For instance, tumor development, microenvironment, treatment strategies and prognosis, are all different in different types of cancer. Thus, we conducted subgroup analysis according to different cancer types. In gastric cancer, no significant heterogeneity existed, and a fixed model was applied in M30 and M65 evaluations. Lower plasma M30 was shown to be a protective factor for gastric cancer patients (HR 0.62, 95% CI 0.38-1.00, *P* =0.05, Figure 3A). However, M65 showed no statistically significant effect in gastric cancer patients' survival (HR 0.48, 95% CI 0.22-1.07, *P* =0.07, Figure 3B). In HCC, a random effect model was applied, and M30 expression had no significant correlation with patient's survival (HR 0.36, 95% CI 0.12-1.09, *P* = 0.07, Figure 3C). However, low M65 level showed to be a protective factor for patients ((HR 0.34, 95% CI 0.20-0.57, *P*< 0.001, Figure 3D. In colorectal cancer, a random effect model was applied, low plasma M30 was a protective factor for patients (HR 0.38, 95% CI 0.15-0.96, *P*=0.04; Figure 3E). However, M65 showed no significant predictive effect in patients' survival (HR 0.40, 95% CI 0.16-1.05, *P*=0.06; Figure 3F). In pancreatic cancer, low M65 showed comparable association with better overall survival in patients and so was a protective factor (HR 0.62, 95% CI 0.41-0.95, *P*=0.03, Figure 3G). We didn't do analysis for M30 in pancreatic cancer because samples are too few.

**Figure 4:**
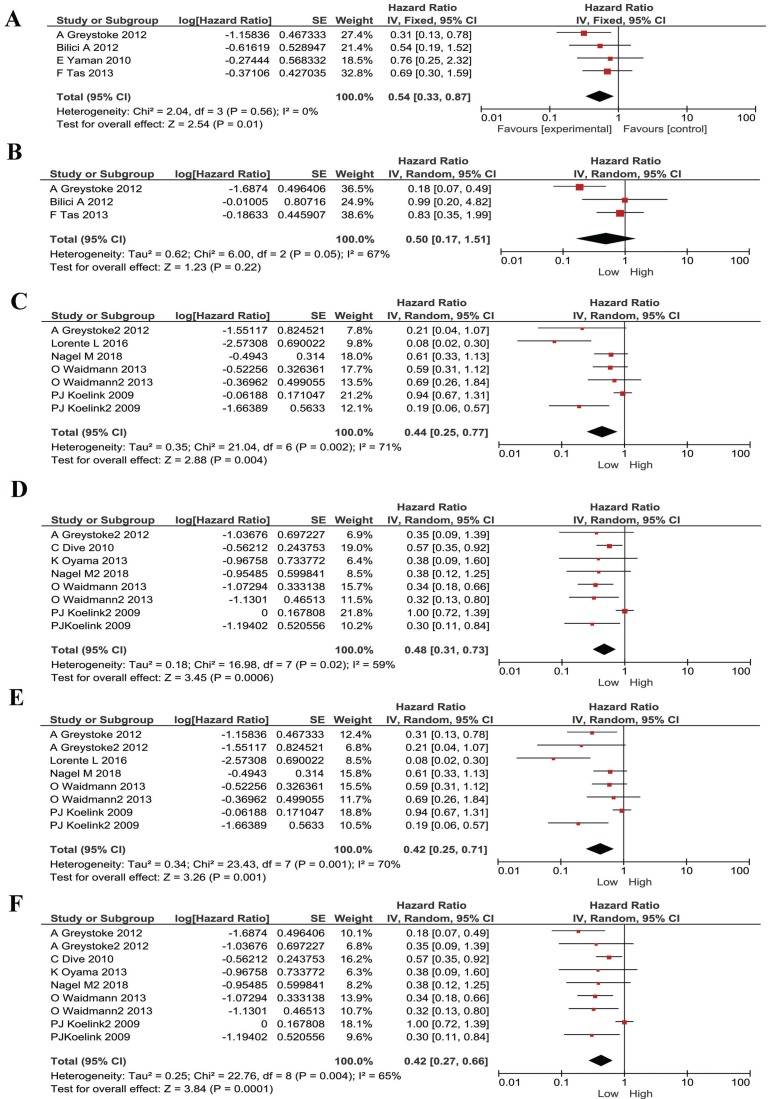
Subgroup analysis for patients stratified by cancer types in M30 and M65 groups. (A) The pooled HRs for M30 in patients with metastasized cancers. (B) The pooled HRs for M65 in patients with metastasized cancers. (C) The pooled HRs for M30 in patients with all-stages cancers. (D) The pooled HRs for M65 in patients with all-stages cancers. (E) The pooled HRs for M30 in Causation patients. (F) The pooled HRs for M65 in Causation patients.

We also noticed that some publications were only studying advanced/ metastasized cancers. Therefore, we divided our included publications into advanced cancer only and all stages. We performed subgroup meta-analyses stratified by cancer stages. The low M30 remained to be a protective factor for metastasized cancer patients (HR 0.54, 95% CI 0.33-0.87, P=0.01, Figure 4A). M65 had no statistically significant correlation with prognosis in advanced cancer patients (HR 0.50, 95% CI 0.17- 1.51, *P*=0.22, Figure 4B). For patients with mixed-stages, lower M30 and low M65 has stronger correlations than the metastasized patients (HR 0.44, 95% CI 0.25-0.77, P=0.004; HR 0.48, 95% CI 0.31-0.73, *P*=0.0006; respectively, Figure 4C and Figure 4D).

Furthermore, since most of our literatures are from west Europe, we did subgroup analyses in these patients (mostly Caucasian). Low M30 and M65 expression remained to be a protective factor for these patients and had same effect (HR 0.42, 95% CI 0.25-0.71, *P*=0.001; HR 0.42, 95% CI 0.27-0.66, P=0.0001; respectively, Figure 4E and Figure 4F).

### Meta-analysis quality control

**Figure 5:**
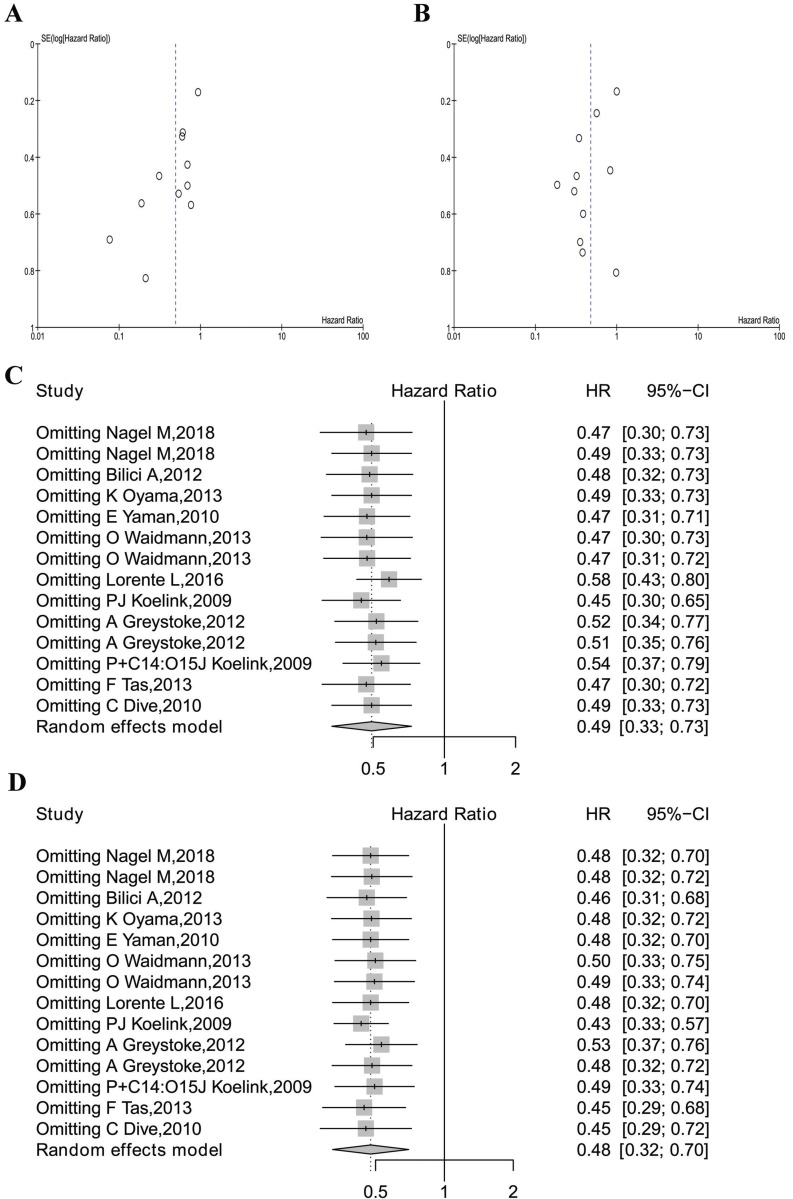
Meta-analysis quality control. (A) The funnel plots of M30 analysis. (B) The funnel plot of M65 analysis. (C) The sensitivity analysis of M30 studies. (D) The sensitivity analysis of all M65 studies.

Begg's funnel test was used to estimate all the existing publication bias of the literature in this meta-analysis. As shown in Figure 5A, the shape of the funnel plots of overall M30 showed evidence of asymmetry, with an Egger's test bias -2.4402 (*P* = 0.0147). The funnel plot of M65 revealed evidence of publication bias, which was confirmed by Egger's test (z=-1.4575, *P* = 0.1450, Figure 5B).

Sensitivity analysis in R was used in this analysis by omitting one study at a time (also called “one-study removed” model). The observed M30 and M65 effects on overall survival were not significantly affected by removing any one of the studies included in this meta-analysis, as is shown in Figure 5C and Figure 5D.

## Discussion

In previous studies, CK18 expression has been found elevated in multiple types of cancers, so did them in serum 29-32. Thus, the non-invasive detection of CK18 in serum provided a good method of cancer diagnosis, monitoring and prognosis prediction. However, many reports are either too small or short of prognosis information. Some of them only reported M65 or M30 and omitted the other one. In this paper, we evaluated both the prognostic value of M65 and M30 ELISA assay in four different types of digestive cancers. They were validated to be good biomarkers in digestive cancers with similar efficiency. Patients with relatively lower level of total and caspase cleaved CK18 were predicted to have better prognosis. This observation is consistent with reports from other types of cancers, including breast cancer, lung cancer and esophageal cancer 31, 33, 34. However, results from immunohistochemistry staining of CK18 in breast cancer samples manifested reverse results 33. In our study, we do not include IHC studies because of limited numbers of them. We only found one paper, showing consistent results as ours. Japanese scholars stated that CK18 expression detected by IHC correlated with poor differentiation, use of neo-adjuvant chemotherapy, advanced stage progression and poor prognosis of esophageal squamous cell carcinoma 29. More studies are warranted.

With regards to the function of CK18 in tumor development and progression, it is still controversial. On one hand, accumulating evidence suggested that Ck18 is involved in PI3K/Akt, Wnt, and MAPK signaling pathways 35. Zhang et al showed that local testicular heat treatment in adult monkeys activate ERK1/2 and Akt kinases with the expression of CK18 in Sertoli cells. After the blockage of ERK activation, CK18 expression was inhibited 36. In pancreatic cancer cells, Ck18 correlated with chymotrypsin C levels and further promoted migration, which is consistent with our results 37. On the other hand, opposite results are reported in breast cancer. The transfection of the CK18 gene into human breast cancer cells caused dramatic regression of their malignancy 38. The biological function of Ck18 might vary in different type of cancers.

Despite the controversy over the biological function of Ck18, accumulating lines of evidence revealed its value in cancer diagnosis and prognosis. Differential pattern of expression might assist accurate pathological diagnosis. With immunohistochemistry, Chen et al found that expression of CK5/6, CK14, and CK17 proteins was increased in squamous cell carcinomas, while increased expression of CK7 and CK18 was observed in adenocarcinomas 39. Furthermore, CK18, as an epithelial cell adhesion molecule, has been utilized as a biomarker in FDA approved circulating tumor cell detection technologies for breast and prostate cancers 40. Although most digestive cancers are adenocarcinoma, levels of M65 and M30 increase as the consequences of cell killing chemotherapies. Doreen et al compared their values in gastrointestinal carcinomas patients before and after receiving chemotherapy. By examining the association of the value changes with clinical responses, they found that cancer patients with a partial response or stable disease revealed a significantly higher increase of cleaved CK-18 during chemotherapy as compared to those with progressive disease 32. Similar results were observed by other scholars 41, 42.These studies showed that the detection of CK18 at different stages of gastrointestinal cancer treatment could serve as a good disease monitoring tool. As shown in our study, M30 and M65 predict poor prognosis in pooled analysis of digestive cancer patients with similar potencies. Also, little efficiency difference is found among different types of digestive cancers. In terms of metastasized or advanced tumors, only M30 showed statistical significance. Our results shed light on the clinical utility of CK18 in predicting cancer prognosis.

There are some limitations in our study. First, all studies used ELISA to detect CK18 expression and there are some limitations of ELISA. ELISA is a fast, scalable, and specific assay. However, the cut-off value is based on clinical statistics and it may not be correct for each individual, leading to false-positive or false-negative. Second, the number of included papers and patient for the meta-analysis is limited. It could be the cause of relatively high heterogeneity in this study. We thrive to include more publications. However, to retain to reliability of the study, we omitted several papers with duplicate patients or low qualities. In addition, CK18 is not a cancer specific biomarker, which is altered in other diseases, such as liver dysfunction, intracerebral hemorrhage and malignant middle cerebral artery infarction 43-45. Finally, ethnicity imbalance is a pity of this study. The incidence rates of digestive cancers are highest in East Asian population, but most of the included patients are Caucasian. More Asian population-based studies are required to confirm our results.

In conclusion, the results of this study showed that non-invasive total and cleaved CK18 level detection by ELISA could be potentially a useful predictor of prognosis of digestive cancer patients.

## Supplementary Material

Supplementary tables.

## Figures and Tables

**Table 1:** The main characteristics of 11 included studies.

Cancer type	First author	Year	Country	Study Period	Stage	Clinical setting	N	Sex(M)	Age years	Follow-up months	cutoff method	M30 cutoff	M30 HRs	M30 LogHRs	M65 cutoff	M65 HRs	M65 LogHRs
GC	Nagel M	2018	Germany	NR	G1-G4	NR	54	67	61 (28-84)	NR	Median	222.52U/L	0.61	-0.21467	NR	NR	NR
	Nagel M	2018	Germany	NR	G1-G5	NR	19	NR	NR	NR	NR	NR	NR	NR	768U/L	0.3849	-0.415
	Bilici A	2012	Turkey	2009-2012	III-IV	advanced GC	31	65	59(30-78)	18(4.5-61) months	Median	400.5U/L	0.54	-0.26761	584.6 U/L	0.99	-0.004
	K Oyama	2013	Japan	NR	I-IV	NR	54	44/54	68(31-80)	26.5 (4.5-40.5)	ROC	NR	NR	NR	199.3 U/L	0.38	-0.42
	E Yaman	2010	Turkey	NR	III-IV	advanced GC	38	63		14(3-24)	ROC	83.8 U/L	0.76	-0.11919	NR	NR	NR
HCC	O Waidmann	2013	Germany	2009-2013	0-6	NR	142	85	59.7 ± 10.4	300 ± 262	ROC	879 U/L	0.593	-0.22695	1587 U/L	0.342	-0.466
	O Waidmann	2013	Germany	2009-2013	0-6	NR	125	79	69.8 ± 7.3	261 ± 225	ROC	887 U/L	0.691	-0.16052	1614 U/L	0.323	-0.491
	Lorente L	2016	Spain	1996-2015	child A-C	NR	135	NR	NR	NR	ROC	384 U/L	0.0763	-1.11748	NR	NR	NR
colorectal	PJ Koelink	2009	Netherlands	1983-1991	Dukes(A-D)	NR	211	56	69(31-90)	NR	Median	7.58U/mg	0.94	-0.02687	209.6U/mg	1	0
	A Greystoke	2012	UK	NR	M	metastatic colorectal cancer	55	43/55	65.0 (57.0-72.0)	median: 27	Tertiles	410U/L	0.314	-0.50307	1190U/L	0.185	-0.733
	A Greystoke	2012	UK	NR	T1-4	NR	66	NR	NR	median: 23	Tertiles	149U/L	0.212	-0.67366	431U/L	0.3546	-0.45
	PJ Koelink	2009	Netherlands	NR	Dukes(A-D)	NR	49	63	68(31-84)	to 8 years	Median	59.1U/L	0.1894	-0.72262	260.5U/L	0.303	-0.519
pancreatic	F Tas	2013	Turkey	2010-2013	M	metastatic	26	54	59(32-80)	31.6 weeks (2.4- 77.9)	Median	293.5U/L	0.69	-0.16115	1230.5U/L	0.83	-0.081
	C Dive	2010	UK	1997 -2010	t1-t4	NR	103	57/103	68 (60-74)	NR	NR	NR	NR	NR	500U/L	0.57	-0.244

Study R = Retrospective, Study P = Prospective cohort study, GC= Gastric cancer, HCC = Hepatocellular carcinoma, NR = Not reported, CI= confidence interval, OS = Overall survival, HR = Hazard ratio. N= Number. Sex M= Male. We set High expression CK18 as HR=1, all these are low HR, in most groups. Lower level of M65 and M30 are protective factors for survival.
