# Supplementary material for: Prognostic value of non-invasive serum Cytokeratin 18 detection in gastrointestinal cancer: a meta-analysis
Source: J Cancer. 2019 Aug 27;10(20):4814–23. doi: 10.7150/jca.31408 (PMC6775513; doi:10.7150/jca.31408)
Supplement: Supplementary file 1 — Supplementary tables. [file jcav10p4814s1.pdf]

Supplementary Table 1. PRISMA NMA Checklist of Items to Include When Reporting A Systematic Review Involving a Network Meta-analysis

Section	Item #	Checklist Item	Reported on Page #
**TITLE**			
Title	1	Identify the report as a systematic review *incorporating a network meta-analysis (or related form of meta-analysis).*	*#1*
**ABSTRACT**			
Structured summary	2	Provide a structured summary	#2
**INTRODUCTION**			
Rationale	3	Describe the rationale for the review in the context of what is already known, including mention of why a network meta-analysis has been conducted.	*#3*
Objectives	4	Provide an explicit statement of questions being addressed, with reference to participants, interventions, comparisons, outcomes, and study design (PICOS).	#3
**METHODS**			
Protocol and registration	5	Indicate whether a review protocol exists and if and where it can be accessed (e.g., Web address); and, if available, provide registration information, including registration number.	#3-5
Eligibility criteria	6	Specify study characteristics (e.g., PICOS, length of follow-up) and report characteristics (e.g., years considered, language, publication status) used as criteria for eligibility, giving rationale.	*#4*
Information sources	7	Describe all information sources (e.g., databases with dates of coverage, contact with study authors to identify additional studies) in the search and date last searched.	#3-4
Search	8	Present full electronic search strategy for at least one database, including any limits used, such that it could be repeated.	#3-4
Study selection	9	State the process for selecting studies (i.e., screening, eligibility, included in systematic review, and, if applicable, included in the meta-analysis).	#4
Data collection process	10	Describe method of data extraction from reports (e.g., piloted forms, independently, in duplicate) and any processes for obtaining and confirming data from investigators.	#4

Supplementary Table 1 continued

Section	Item #	Checklist Item	Reported on Page #
Data items	11	List and define all variables for which data were sought (e.g., PICOS, funding sources) and any assumptions and simplifications made.	#4
Geometry of the network	S1	Describe methods used to explore the geometry of the treatment network under study and potential biases related to it. This should include how the evidence base has been graphically summarized for presentation, and what characteristics were compiled and used to describe the evidence base to readers.	*#5*
Risk of bias within individual studies	12	Describe methods used for assessing risk of bias of individual studies (including specification of whether this was done at the study or outcome level), and how this information is to be used in any data synthesis.	*#5*
Summary measures	13	State the principal summary measures (e.g., risk ratio, difference in means).	*#5*
Planned methods of analysis	14	Describe the methods of handling data and combining results of studies for each network meta-analysis.	*#5*
Assessment of Inconsistency	S2	Describe the statistical methods used to evaluate the agreement of direct and indirect evidence in the treatment network(s) studied. Describe efforts taken to address its presence when found.	*#5*
Risk of bias across studies	15	Specify any assessment of risk of bias that may affect the cumulative evidence (e.g., publication bias, selective reporting within studies).	*#5*
Additional analyses	16	Describe methods of additional analyses if done, indicating which were pre-specified.	*#5*
**RESULTS†**			
Study selection	17	Give numbers of studies screened, assessed for eligibility, and included in the review, with reasons for exclusions at each stage, ideally with a flow diagram.	*#5*
Study characteristics	18	For each study, present characteristics for which data were extracted (e.g., study size, PICOS, follow-up period) and provide the citations.	#6 and Table1
Risk of bias within studies	19	Present data on risk of bias of each study and, if available, any outcome level assessment.	#7
Results of individual studies	20	For all outcomes considered (benefits or harms), present, for each study: 1) simple summary data for each intervention group, and 2) effect estimates and confidence intervals.	*#6-#7*

Supplementary Table 1 continued

Section	Item #	Checklist Item	Reported on Page #
Synthesis of results	21	Present results of each meta-analysis done, including confidence/credible intervals. If additional summary measures were explored (such as treatment rankings), these should also be presented.	*#6-#7*
Risk of bias across studies	22	Present results of any assessment of risk of bias across studies for the evidence base being studied.	*#7*
Results of additional analyses	23	Give results of additional analyses, if done.	*#6-#7*
**DISCUSSION**			
Summary of evidence	24	Summarize the main findings, including the strength of evidence for each main outcome; consider their relevance to key groups (e.g., healthcare providers, users, and policy-makers).	#7
Limitations	25	Discuss limitations at study and outcome level (e.g., risk of bias), and at review level (e.g., incomplete retrieval of identified research, reporting bias).	#9
Conclusions	26	Provide a general interpretation of the results in the context of other evidence, and implications for future research.	#9
**FUNDING**			
Funding	27	Describe sources of funding for the systematic review and other support (e.g., supply of data); role of funders for the systematic review. This should also include information regarding whether funding has been received from manufacturers of treatments in the network and/or whether some of the authors are content experts with professional conflicts of interest that could affect use of treatments in the network.	*#10*

PICOS = population, intervention, comparators, outcomes, study design.

Supplementary Table 2. Quality assessment of 11 full-text studies included in the meta-analysis according to predefined nine items.

First author	Year	A clear description of the objectives	A clear ethical statement	Study period	A clear description of tumor stage	A patient selection/exclusion criterion	Stating the cutoff of CK18	Pre-define survivals	Multivariate analysis and/or univariate analysis	A clear HRs with 95% CI	Limitations considered in the study	Score
Nagel M	2018	Yes	Yes	No	Yes	No	Yes	No	Yes	No	No	5
Nagel M	2018	Yes	Yes	No	Yes	No	No	No	Yes	No	No	4
Bilici A	2012	Yes	Yes	Yes	Yes	Yes	Yes	No	Yes	Yes	Yes	9
K Oyama	2013	Yes	Yes	No	Yes	Yes	Yes	No	Yes	Yes	No	7
E Yaman	2010	Yes	Yes	No	Yes	Yes	Yes	No	Yes	No	Yes	7
O Waidmann	2013	Yes	Yes	Yes	Yes	Yes	Yes	Yes	Yes	Yes	No	9
O Waidmann	2013	Yes	Yes	Yes	Yes	Yes	Yes	Yes	Yes	Yes	No	9
H Elalfy	2018	Yes	Yes	Yes	Yes	Yes	Yes	No	Yes	No	Yes	8
Lorente L	2016	Yes	No	Yes	Yes	No	Yes	No	No	Yes	Yes	6
PJ Koelink	2009	Yes	Yes	Yes	Yes	No	Yes	No	Yes	Yes	No	7
A Greystoke	2012	Yes	Yes	No	Yes	No	Yes	No	Yes	Yes	Yes	7
A Greystoke	2012	Yes	Yes	No	Yes	No	Yes	No	Yes	Yes	Yes	7
PJ Koelink	2009	Yes	Yes	No	Yes	No	Yes	No	Yes	Yes	Yes	7
F Tas	2013	Yes	No	Yes	Yes	No	Yes	No	Yes	No	Yes	6
C Dive	2010	Yes	No	Yes	Yes	No	No	No	Yes	No	No	4
